# A collection of rumen bacteriome data from 334 mid-lactation dairy cows

**DOI:** 10.1038/sdata.2018.301

**Published:** 2019-01-22

**Authors:** Hui-Zeng Sun, Mingyuan Xue, Le Luo Guan, Jianxin Liu

**Affiliations:** 1 Department of Agricultural, Food & Nutritional Science, University of Alberta, Edmonton, AB, T6G 2P5, Canada; 2 Institute of Dairy Science, MoE Key Laboratory of Molecular Animal Nutrition, College of Animal Sciences, Zhejiang University, Hangzhou, 310058, P.R. China

**Keywords:** Metagenomics, Microbiome, Next-generation sequencing

## Abstract

With the help of the bacteria in the rumen, ruminants can effectively convert human inedible plant fiber to edible food (meat and milk). However, the understanding of rumen bacteriome in dairy cows is still limited, especially in a large population under the same diet, breed, and milking period. Here we described the sequencing data of 16S rRNA gene of rumen bacteriome from 334 mid-lactation Holstein dairy cows generated using the Illumina HiSeq 2500 (PE250) platform. A total of 24,030,828 raw reads with an average of 71,946 ± 13,450 sequences per sample were obtained. The top ten genera with highest relative abundance accounted for 60.65% of total bacterial sequences. We observed 4,460 overall operational taxonomic units (1,827 ± 94 per sample) based on a 97% nucleotide sequence identity between reads. Totally 6,082 amplicon sequence variants (672 ± 131 per sample) were identified in 334 samples. The shareable datasets can be re-used by researchers to assess other rumen bacterial-related biological functions in dairy cows towards the improvement of animal production and health.

## Background & Summary

Dairy cows play important roles in supplying milk to humans and harnessing solar energy by efficiently converting plant biomass to nutrients that are absorbed and utilized by animals to produce milk^1^. This process is mainly attributed to the ruminal microbiota, especially to the bacterial community. Bacteria are the predominant microbes in the rumen (>91% of the whole microbiome^2^) who produce volatile fatty acids and microbial protein that provide more than 70% of required energy^3^ and 60% of non-ammonia nitrogen^4^ to the dairy cow. It is well known that the composition of ruminal bacteriome of the dairy cow is highly affected by diet, age, geographic location, season, feeding cycle, and feeding regimen^5^, as well as host animal (even varies under the same dietary condition)^6^. The evidence has shown that ruminal bacterial population is associated with milk production and milk composition in dairy cows^7–9^, because they are tightly linked to cows’ ability to harvest energy from feed^10^. However, few consistent specific conclusions can be drawn from these reported results because of the variables in breeds, diet, milking period, sampling size, and so on.

The 16S rRNA gene amplicon sequencing has become an important method to study the composition of bacterial communities in environmental samples^11,12^. Most of the previous studies of rumen bacteriome using 16S rRNA gene amplicon sequencing were based on Illumina MiSeq platform^13^. With the continuous development of high-throughput sequencing techniques, the upgraded Illumina HiSeq platform enables achieving 2 × 250 bp paired-end (PE250) reads, which presents the same reads length but much higher throughput and sequencing quality than MiSeq^14,15^. With the advantages of high sequencing depth, accurate identification of low-rich species, and improvements on the integrity of microbial community, the HiSeq PE250 has its potential to become the prioritized choice of 16S rRNA gene amplicon sequencing-based microbial community study in the dairy cow^16^.

**Figure 1:**
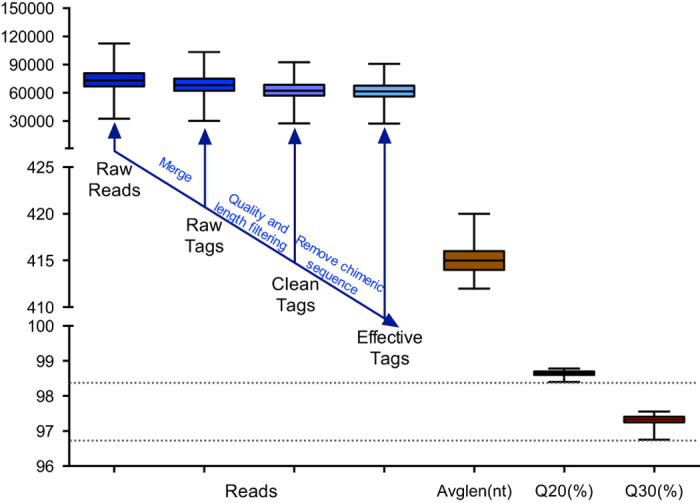
The reads output of sequencing data. Q20 and Q30 refer to the percentage of bases with the quality score greater than 20 (sequencing error rate less than 1%) and 30 (sequencing error rate less than 0.1%) in the effective tag, respectively.

In this study, the collection of rumen bacteriome data was performed from a large cohort of dairy cows (334 individuals) using Illumina HiSeq 2500 (PE250) based 16S rRNA gene amplicon sequencing of the V3-V4 region. All the cows were Holstein dairy cow, which is known as the most popular and highest-productive dairy animals worldwide^17^. A total of 24,030,828 raw reads were generated, with an average of 71,946 ± 13,450 sequences per sample (Fig. 1). After sequencing data processing including reads split, data filtering, and chimera removal (see methods), an average of 67,014 ± 12,396 raw tags, 61,370 ± 11,165 clean tags, 60,429 ± 10,963 effective tags were obtained (Fig. 1). The average length of effective tags was 415.21 ± 1.53 nucleotides (nt). The percentage of bases in the effective tags with a phred quality score of 20 or higher (predicted to have an accuracy of 99% or higher) and with a phred quality score of 30 or higher (predicted to have an accuracy of 99.9% or higher) were 98.64 ± 0.07% and 97.31 ± 0.15%, respectively (Fig. 1). The raw reads files and phenotypic data were released with our previous paper (Data Citation 1), which suggested that the pan and core rumen bacteriome potentially contribute to variations of milk production traits^18^.

**Figure 2:**
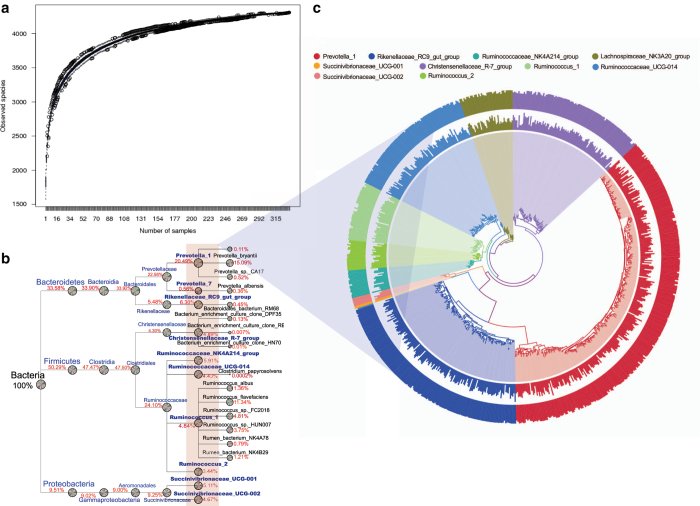
The species accumulation boxplot and phylogenetic relationships. (**a**) The species accumulation boxplot. The x-axis represents the number of samples, and the y-axis represents the number of identified OTUs. (**b**) The taxonomy tree generated from all the samples from kingdom to species levels. Only the top 10 most abundant genera related results were displayed. The average percentage of each taxa based on the total bacterial sequencing reads at different levels from 334 samples was labeled. The piechart with different colors within the circle indicates different samples. (**c**) OTU cluster tree under the top 10 most abundant genera. OTU: operational taxonomic unit.

The overall number of operational taxonomic units (OTUs) reached 4,460 based on a 97% nucleotide sequence identity with an average of 1,827 ± 94 OTUs per rumen sample (Fig. 2a). Sample-based species accumulation boxplot showed the OTU numbers increased as a function of the number of samples. The curve became asymptotically stable along with the OTU number saturated and an increasing smaller number of new OTUs were added in each sample (Fig. 2a), indicating adequate sequencing depth to represent rumen bacterial composition accurately (with the Good’s coverage > 99.9%). The top 10 genera with the highest relative abundance were *Prevotella_1* (20.49%, 274 OTUs), *Prevotella_7* (0.56%, 8 OTUs), *Rikenellaceae_RC9_gut_group* (6.30%, 150 OTUs), *Christensenellaceae_R-7_group* (4.89%, 100 OTUs), *Ruminococcaceae_NK4A214_group* (5.91%, 20 OTUs), *Ruminococcaceae_UCG-014* (4.43%, 93 OTUs), *Ruminococcus_1* (4.84%, 43 OTUs)*, Ruminococcus_2* (3.44%, 22 OTUs), *Succinivibrionaceae_UCG-001* (5.11%, 2 OTUs), and *Succinivibrionaceae_UCG-002* (4.67%, 6 OTUs) (Fig. 2b and c), which accounted for 60.65 ± 5.20% (mean ± SD) of total bacterial sequences and belonged to the most abundant three phyla: *Firmicutes* (50.29%), *Bacteroidetes* (33.58%) and *Proteobacteria* (9.51%). A total of 6,082 amplicon sequence variants (ASVs) were identified in 334 samples (at least one time certain ASV occurred in one sample) with an average of 672 ± 131 ASVs per rumen sample (ASV_table, Data Citation 2).

Herein, we provided the description of up-to-now largest numbers of rumen bacteriome samples in the mid-lactation Holstein dairy cow, related phenotypes, and detailed methods for identification and validation of 16S rRNA gene sequencing reads. These data will be a valuable resource for microbiology, and can be shared and re-used by the research community to investigate other questions on rumen microbiology in dairy cow towards the improvement of animal production and health.

## Methods

The experimental procedures were approved by the Animal Care and Use Committee of Zhejiang University (Hangzhou, China) in compliance with the University’s guidelines for animal research. The brief descriptions of material and method were reported in our previous work^18^. Here we described either complete or new supplementary details where necessary.

### Animals and phenotypes

A total of 334 Holstein dairy cows in the mid-lactation period (days in milk = 159 ± 34, mean ± SD) were used in this study. All the animals were raised under the same management conditions, fed the same diet as total mixed ration (Diet_ingredient, Data Citation 2) with a concentrate-to-forage ratio of 57:43 (DM basis), and had free access to water. The phenotypes including parity of the cows, rumen pH, concentrations of ammonia-nitrogen and volatile fatty acids (acetate, propionate, butyrate, isobutyrate, valerate, and isovalerate) in the rumen, and milk performance (daily milk yield, milk contents of protein, fat and lactose, and milk urea nitrogen) were recorded.

Rumen fluid samples were collected using the oral stomach tube (OST, Anscitech Co. Ltd., Wuhan, China), which was inserted into the central rumen (~200 cm depth) in order to get most representative samples^19^. The first 150 mL of ruminal fluid was discarded to avoid saliva contamination. Rumen samples were snap-frozen in liquid nitrogen and subsequently stored at −80 °C until further analysis. Between samples, OST was rinsed and protective gloves were replaced to prevent crossed contamination.

### Genomic DNA extraction

The total DNA of rumen sample was extracted using a bead-beating method according to the published paper^20^. Briefly, about 1 g of rumen samples were transferred into a 10-mL tube after thawed on the ice. With the addition of 4.5 mL of TN150 buffer (10 mM Tris HCl (pH 8.0, 150 mMNaCl), the mixture was vortexed for 30 S vigorously and subjected for centrifugation at 4°C, 200 × g for 5 min. The upper phase (1 mL) was transferred into a 2-mL microcentrifuge tube, then 0.3 g of sterile Zirconium beads (diameter, 0.1 mm) was added and centrifuged at 4°C, 14,600 × g for 5 min. The pellet was resuspended in 1 mL of TN150 buffer after discarded the supernatant and placed in a mini BeadBeater (Bio Spec Products Inc., Bartlesville, USA) at 480 rpm for 3 min. Following the extraction with phenol, chloroform-isoamyl alcohol (24:1), DNA was precipitated using cold ethanol at −20 °C for 4 h and dissolved in 60 μL of nuclease-free TE buffer (10 mM Tris HCl (pH 8.0), 1 mM EDTA). The DNA concentration was measured using the NanoDrop 2000 spectrophotometer (NanoDrop Technologies, Wilmington, USA) and DNA purity were assessed with 1% agarose gel eletrophoresis (100 v, 40 min). Based on the concentration, DNA sample was diluted to 50 ng/μL using TE buffer for further processing.

### Amplicon generation

The amplicon of the V3-V4 hypervariable region of 16S rRNA genes was performed using the primer set 341 F/806 R (341 F: 5′-CCTAYGGGRBGCASCAG-3′; 806 R: 5′-GGACTACNNGGGTATCTAAT-3′)^21^ and 6-bp error-correcting barcode at the 5′ terminus of reverse primer that unique to each DNA sample. The PCR reaction solution consisted of 0.5 U of Taq polymerase (TransGen Biotech Co., Ltd., Beijing, China) in a 25 μl of 10 × PCR reaction Buffer, 200 μM of each dNTP, 0.2 μM of each primer and 2 μl of DNA. Thirty-five cycles PCR reactions were carried out using Phusion High-Fidelity PCR Master Mix (New England Biolabs Ltd., Ipswich, USA) with GC buffer and high efficiency-high fidelity enzyme to ensure the efficiency and accuracy of amplification^11^, which was done with the following procedures: 1) at 94 °C for 3 min; 2) 35 cycles at 94 °C for 45 s, 50 °C for 60 s and 72 °C for 90 s; 3) final extension at 72 °C for 10 min. The PCR products were mixed with the same volume of 1 × loading buffer (contained SYBR safe) and conducted electrophoresis detection on 2% agarose gel (80 v, 40 min). Sample with a bright band between 400–450 bp was used for library construction.

### Library construction and sequencing

Before library preparation, the PCR products were mixed in equimolar ratio and purified using Qiagen Gel Extraction Kit (Qiagen, Hilden, Germany). Sequencing libraries were constructed using TruSeq DNA PCR-Free Sample Preparation Kit (Illumina Inc., San Diego, USA) according to the manufacturer’s instructions. The library quality was assessed by the Qubit 2.0 Fluorometer (Thermo Fisher Scientific Inc., Waltham, USA) and Agilent Bioanalyzer 2100 system (Agilent Technologies Inc., Santa Clara, USA). The library was sequenced on an Illumina HiSeq 2500 platform based on standard protocol^22^ by Novogene Bioinformatics Technology Co. Ltd. (Tianjin, China) to generate paired-end reads (2 × 250 bp).

### Sequencing data analysis

The sequencing data analysis consisted of reads split, sequence assembly, data filtering, and chimera removal. Paired-end reads were assigned into samples to get the raw reads of each sample based on their unique barcode and truncated by cutting off the barcode and primer sequence. Raw reads of each sample were joined into single sequence based on overlapping regions to get the raw tags (splicing sequencing) using Fast Length Adjustment of Short Reads (version 1.2.7, http://ccb.jhu.edu/software/FLASH/), which was an accurate and efficient analysis tool and designed to merge paired-end reads when at least some of the reads have overlapped with the reads generated from the opposite end of the same DNA fragment^23^. Data filtering of the raw tags was performed to obtain high-quality clean tags^24^ based on the quality control process of Quantitative Insight Into Microbial Ecology (QIIME, version 1.7.0, http://qiime.org/index.html)^25^ with the following conditions: 1) Tag truncation: the raw tag was truncated from the first low-quality base site where the number of continuous low-quality bases (quality score < 20) reached to the set length (default value = 3); and 2) Length filtering: to delete the tags with continuous high quality (phred quality score ≥20) base length less than 75% of the tag length. In the chimera removal step, the clean tags were compared with the reference database (Gold database, http://drive5.com/uchime/uchime_download.html) using UCHIME algorithm in Usearch v11 (http://www.drive5.com/usearch/manual/uchime_algo.html)^26^ to identify chimera sequences and remove the chimera sequences^27^.

### OTUs cluster and taxonomic annotation

Sequences in effective tags with identity greater than 97% were assigned to the same OTUs using the UPARSE (version7, http://drive5.com/uparse/)^28^. The most abundant sequences in each OTU were defined as representative sequences and were conducted for taxonomic annotation in each level (phylum, class, order, family, genus, and species) against the GreenGene database13.8^29^ based on Ribosomal Database Project classifier (http://sourceforge.net/projects/rdp-classifier/)^30^. Some powerful microbiome data analysis platform enable comprehensive down-stream and co-processing analysis starting with OTU tables^31^, for example, the marker gene data profiling (composition and diversity analysis, comparative analysis, and prediction of metabolic potentials) and projection with public data analysis (co-processing data together with a suitable public 16S rRNA data of interest and explore the results) are available in the MicrobiomeAnalyst (http://www.microbiomeanalyst.ca/).

Sample-based species accumulation boxplot and rarefaction curve were generated to testify sequencing depth for providing sufficient OTU coverage to describe the bacterial composition accurately^32^. To further study the phylogenetic relationships among different OTUs, multiple sequence alignment was performed using the MUSCLE (http://www.drive5.com/muscle/)^33^ and displayed by iTOL (version 4, https://itol.embl.de/)^34^. Good’s coverage of counts was calculated to represent the sequencing depth, which is defined as 1−F_1_/N, where F_1_ is the number of singlet on OTUs and N is the total number of individuals (sum of abundances for all OTUs).

### Amplicon sequence variants analysis

To improve the precision, reusability, comprehensiveness and reproducibility of marker-gene data analysis^35^, a higher-resolution ASVs analysis were performed using R software (version 3.5.1) based on DATA2 pipeline (package version 1.8.0, https://benjjneb.github.io/dada2/tutorial.html). The demultiplexed fastq files (one forward and one reverse) of each samples without non-biological nucleotides (e.g. primers, adapters) were used to generate ASVs table, which presented the number of times each exact amplicon sequence variant observed in each sample. Default parameters in DATA2 pipeline tutorial (1.8) were applied in ASVs analysis, in which trimmed the forward reads at position 240, and the reverse reads at position 160, filtered out all reads with more than 0 ambiguous nucleotides and 2 expected errors.

## Data Records

The raw reads files (fastq format) of each sample have been uploaded to the NCBI Sequence Read Archive (SRA). All data can be used without restrictions. Additional datasets including clean reads files (fastq format) of each samples (Data citation 2), OTU annotation table (OTU_table, Data citation 2), multiple-sequence alignment table at phylum (Taxonomy_phylum, Data citation 2) and genera level (Taxonomy_genera, Data citation 2), individual measurements of phenotypic data (Phenotypes, Data citation 2), ASVs table were submitted to the integrated figshare system.

## Technical Validation

**Figure 3:**
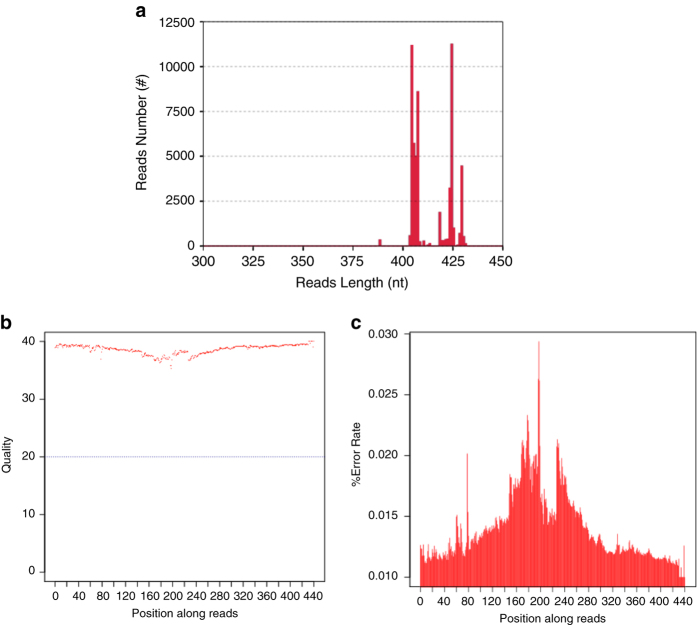
The quality assessment of sequencing data. (**a**) The length distribution of merged reads. (**b**) The quality score distribution of sequencing data. (**c**) The error rate distribution of sequencing reads.

The qualified genomic DNA (total amount ≥1 μg, concentration ≥50 ng/μL, and 1.8 <OD_260_/280 < 2.0) were subjected for amplicon generation. The sequencing library quality was checked by the Qubit 2.0 Fluorometer (Thermo Fisher Scientific Inc., Waltham, USA) and Agilent Bioanalyzer 2100 system (Agilent Technologies Inc., Santa Clara, USA). The libraries with qualified concentration (≥5nM) and volume (>5 μL) were subjected for sequencing. The quality of sequencing data was assessed by the length distribution of merged reads, quality distribution of sequencing data, and error rate distribution of sequencing reads. More than 99% of merged reads had the length of 400–430 nt (Fig. 3a). The sequencing data with the quality score greater than 30 accounted for 97% of all the effective tags (Fig. 3b). The error rate of sequencing reads showed relatively higher in the ending position but presented a low level entirely (<0.3%) (Fig. 3c). Data filtering was used for sequencing data pre-processing with the parameters of minimum quality score ≥20, and read length with continuous high-quality bases ≥75% of tag length.

## Additional information

**How to cite this article**: Sun, H. Z. *et al*. A collection of rumen bacteriome data from 334 mid-lactation dairy cows. *Sci. Data*. 6:180301 doi: 10.1038/sdata.2018.301 (2019).

**Publisher’s note**: Springer Nature remains neutral with regard to jurisdictional claims in published maps and institutional affiliations.

## Supplementary Material
